# Association of long-term blood pressure variability and brachial-ankle pulse wave velocity: a retrospective study from the APAC cohort

**DOI:** 10.1038/srep21303

**Published:** 2016-02-19

**Authors:** Yang Wang, Yuling Yang, Anxin Wang, Shasha An, Zhifang Li, Wenyan Zhang, Xuemei Liu, Chunyu Ruan, Xiaoxue Liu, Xiuhua Guo, Xingquan Zhao, Shouling Wu

**Affiliations:** 1 Graduate School, North China University of Science and Technology, Tangshan, China; 2 Department of Neurology, Beijing Tiantan Hospital, Capital Medical University, Beijing, China; 3 China National Clinical Research Center for Neurological Diseases, Beijing, China; 4 Center of Stroke, Beijing Institute for Brain Disorders, Beijing, China; 5 Beijing Key Laboratory of Translational Medicine for Cerebrovascular Disease, Beijing, China; 6 Department of Epidemiology and Health Statistics, School of Public Health, Capital Medical University, Beijing, China; 7 Department of Cardiology,Tangshan People’s Hospital, Tangshan, China; 8 Department of Cardiology, Kailuan Hospital, North China University of Science and Technology, Tangshan, China

## Abstract

We investigated associations between long-term blood pressure variability (BPV) and brachial-ankle pulse wave velocity (baPWV). Within the Asymptomatic Polyvascular Abnormalities Community (APAC) study, we retrospectively collected long-term BPV and baPWV measures. Long-term BPV was calculated using the mean and standard deviation of systolic blood pressure (SBP) across 4 years based on annual values of SBP. In total, 3,994 subjects (2,284 men) were eligible for inclusion in this study. We stratified the study population into four SBP quartiles. Left and right baPWV was higher in participants with long-term SBPV in the fourth quartile compared with the first quartile (left: 1,725 ± 488 vs. 1,461 ± 340 [p < 0.001]; right: 1,722 ± 471 vs. 1,455 ± 341 [p < 0.001], respectively). We obtained the same result for total baPWV (fourth vs. first quartile: 1,772 ± 429 vs. 1,492 ± 350 [p < 0.001]). Furthermore, there was a trend for gradually increased baPWV (≥1,400 cm/s) with increased SBPV (p < 0.001). After multivariable adjustment, baPWV was positively correlated with long-term BPV (p < 0.001). In conclusion, long-term BPV is significantly associated with arterial stiffness as assessed by baPWV.

Blood pressure variability (BPV) is estimated using the standard deviation of beat-to-beat BP obtained by intra-arterial monitoring for 24 h^1^ or the standard deviation of non-invasive ambulatory BP monitoring^2^,^3^. BPV is classified into two types: short-term BPV, which fluctuates across 24 h (beat-to-beat, minute-to-minute, hour-to-hour, and day-to-night changes); and long-term BPV, which fluctuates over more-prolonged periods (days, weeks, months, seasons, and even years)^4^. Increased short-term and long-term BPV are related to the development, progression, and severity of cardiac, vascular, and renal damage, and linked to increased risk of cardiovascular morbidity and mortality^2^,^5^. Research suggests higher long-term BPV is related to more severe target organ damage, thicker carotid intima-media, left ventricle hypertrophy, transient ischemic attack, and micro-albuminuria^6^. Furthermore, Rothwell *et al.*^5^ observed that long-term BPV is a strong predictor for cardiovascular events independent of systolic blood pressure (SBP).

The pathological basis of target organ damage is changes in artery structure and functions. Pulse wave velocity (PWV) is a surrogate marker for arterial stiffness. Brachial-ankle pulse wave velocity (baPWV) is a relatively simple, non-radiating, non-invasive and readily available measure of central and peripheral arterial stiffness, used widely in Asian countries^7^. It provides similar values to carotid–femoral PWV, heart–ankle PWV^8^, and aortic PWV^9^ measured using invasive catheter methods. Several studies have demonstrated that increased PWV predicts cardiovascular morbidity and mortality in hypertensives^10^,^11^,^12^ and is significantly connected with SBP^13^ and pulse pressure^14^.

Therefore, in this prospective cohort study, we calculated the correlation between BPV and baPWV, using the Asymptomatic Polyvascular Abnormalities Community (APAC) study database.

## Methods

### Study Design and Population

The APAC study is an observational, prospective, community-based study investigating the epidemiology of asymptomatic polyvascular abnormalities in Chinese adults. The study was approved by the Beijing Tiantan Hospital and Ethics Committee of Kailuan General Hospital, in compliance with the Declaration of Helsinki. All participants signed written informed consent. The APAC study enrolled a subpopulation of the Kailuan study, a prospective cohort study investigating risk factors for chronic diseases (such as myocardial infarction, stroke, and cancers) in the Kailuan community in Tangshan, Hebei Province^15^. All 155,418 residents in the Kailuan community were invited to participate in the Kailuan study, including routine blood, urine, and biochemical tests every 2 years. Among them, 65.3% agreed and signed written informed consent and a total of 101510 participants (81 110 men, 18–98 years) were recruited in the Kailuan study in 2006. Using stratified random sampling by age and sex, 7,000 subjects older than 40 years were randomly selected between June 2010 and June 2011 from the Kailuan cohort. A total of 5,852 people agreed to participate in the APAC study and 5,816 subjects eventually completed the baseline data collection. Of these, 376 people did not meet the following inclusion criteria: (1) no history of transient ischemic attack, stroke, or coronary disease as assessed at baseline; and (2) absence of neurologic deficits indicating previous stroke. As such, 5,440 cases were enrolled.

The questionnaire was completed by specially trained investigators who interviewed participants face to face. The questionnaire assessed demographic data, occupational status, behavior and habits (sleep, smoking, drinking, physical activity and diet), medical history, and family history. Smokers were defined as those smoking a daily average of at least one cigarette, lasting nearly one year. Alcohol abuse was defined as an alcohol intake of at least 45 g of liquor a day for more than one year for women and at least 90 g of liquor a day for more than one year for men. We categorized physical activity as very active (exercise > 80 min/week), moderately active (exercise: 1 to 80 min/week) or inactive (no exercise).

Participants were requested not to smoke or drink tea or coffee for 30 minutes, and to sit quietly for 15 minutes, before blood pressure was measured. We measured right brachial artery blood pressure using a calibrated mercury sphygmomanometer three times, with each measurement separated by a 1 to 2 minute interval. We then took the average value as the final blood pressure. All blood samples were analyzed at the Kailuan General Hospital central laboratory using a Hitachi 747 auto-analyzer (Hitachi, Tokyo, Japan). We measured triglyceride (TG), total cholesterol (TC), low density lipoprotein cholesterol (LDL-C), high density lipoprotein cholesterol (HDL-C), fasting blood glucose (FBG), high sensitivity C reactive protein (Hs-CRP), and other related laboratory indices. Baseline characteristics and details of the APAC study design have been published previously^16^,^17^.

### Assessment of baPWV

We collected baPWV values using a BP-203 RPE III networked arteriosclerosis detection device produced by Omron health medical (China) Co., LTD. Measurements were taken between 7:00 and 9:00 in the morning on the examination day. Using the network connection, we could read the data directly. During BaPWV assessment, participants sat for more than 5 minutes in a room with a controlled temperature of 22 °C to 25 °C. Smoking and drinking of coffee, tea or alcohol were prohibited for at least 3 hours and exercise was prohibited for 30 minutes before the measurement of baPWV. Participants wore light clothing, laying in a supine position without a pillow and were asked to keep quiet during the examination. Cuffs were wrapped on both arms and ankles. The lower edge of the arm cuff was positioned 2–3 cm above the cubital fossa transverse striation, while the lower edge of the ankle cuff was positioned 1–2 cm above the medial malleolus. The heartbeat monitor was placed on the left edge of the sternum, and electrocardiogram electrodes were placed on both wrists. BaPWV was measured twice in each participant in both 2010 and 2012. Data from 2012 were used for the analyses. Moreover, to validate our findings, we repeated the statistical analysis using the baPWV value from 2010. The higher value of the left side and right side was used for the total value. According to the standard of the American Heart Association medical/scientific statement (1993), baPWV values higher than 1,400 cm/s were judged to indicate peripheral arteriosclerosis. The method of measuring baPWV was the same in 2010 and 2012.

### Calculation of long-term BPV

As there is a greater correlation between SBPV and target organ damage than between diastolic blood pressure variability and target organ damage^18^,^19^, we analyzed the results using inter-annual SBPV values. Currently, the calculation of long-term BPV uses degree of variation of blood pressure. The SD from the four averaged annual SBP values was regarded as the index of SBPV for each subject, and their group mean and SD values were presented as the SBPV results for each SBPV group blood pressure.

### Statistical analysis

Statistical analysis was performed using a commercially available software program (SAS software version 9.3, SAS Institute Inc., Cary, North Carolina, USA). As there were no clear cut points for the SBPV measures, we stratified the study population into quartiles. Continuous variables were described by their means ± standard deviations and categorical variables were described as percentages. As TG and Hs-CRP were not normally distributed, log10 transformations were used. We used ANOVA to compare non-paired samples of normally distributed parameters and the Kruskal-Wallis test for the comparison of nonparametric variables. The chi-square test was applied to compare categorical variables. We used multivariate linear regression to analyze the correlation between long-term SBPV and baPWV. To systematically adjust for all potential confounders, four models were used. Model 1 was the unadjusted model. Model 2 included age and sex as independent variables. Model 3 further adjusted for age, sex, smoking, drinking, physical activity, and body mass index. In addition to the independent variables analyzed in model 3, model 4 included SBP, total cholesterol, triglyceride, low density lipoprotein cholesterol, and fasting blood glucose. Two-sided p-values are reported for all analyses. Differences are statistically significant where p < 0.05. We used the average SBP to replace missing values for 2006, 2008, 2010 and 2012. We conducted a sensitivity analysis by repeating the above statistical analysis using complete data for all 4 years, avoiding the impact of interpolated data.

## Results

**Figure 1:**
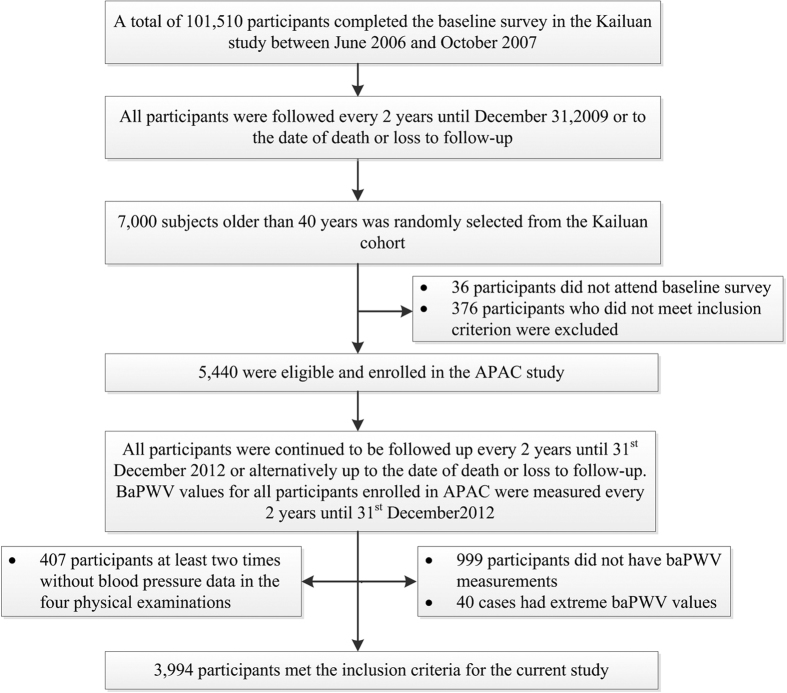
Flowchart of the study. APAC: Asymptomatic Polyvascular Abnormalities Community; baPWV: brachial-ankle pulse wave velocity.

The APAC study enrolled 5,440 subjects (40.1% women). For this study, we excluded 407 participants at least two times without blood pressure data in the four physical examinations, 999 participants without baPWV measurements, and 40 cases with extreme baPWV values. Eventually, 3,994 subjects (2,284 men) were eligible for inclusion in this study. (Fig. 1)

Table 1 provides demographic and baseline clinical characteristics of subjects in different SBPV groups. The mean age was 50.57 ± 10.90 years (range: 40–88 years). Mean SBPV was 10.93 ± 6.01 mmHg. We stratified participants into four groups, according to SBPV quartiles: quartile 1 (SBPV < 6.57 mmHg), quartile 2 (6.57 ≤ SBPV < 9.74 mmHg), quartile 3 (9.74 ≤ SBP < 14.04 mmHg), and quartile 4 (SBPV ≥ 14.04 mmHg). The mean of SBPV for each SBPV group were 4.57 ± 1.60 mmHg, 8.36 ± 0.90 mmHg, 11.73 ± 1.25 mmHg, 19.03 ± 5.15 mmHg; respectively. Participants in quartile 4 were more likely to be older and have a higher SBP. Different SBPV groups differed significantly by sex, body mass index (BMI), TG, TC, FBG, physical activity, and Hs-CRP (p < 0.05). Higher left and right baPWV was more common in participants with long-term SBPV in the fourth quartile compared with the first quartile (left side: 1,725 ± 488 vs. 1,461 ± 340 [p < 0.001]; right side: 1,722 ± 471 vs. 1,455 ± 341 [p < 0.001], respectively). We observed similar results for total baPWV (fourth vs. first quartile: 1,772 ± 429 vs. 1,492 ± 350 [p < 0.001]). Furthermore, there was a trend for gradually increased baPWV (≥1400 cm/s) with increased SBPV (p < 0.001).

There was a positive correlation between SBPV and baPWV (r = 0.245, p < 0.001). A multivariate linear regression was performed to investigate the association between long-term BPV and baPWV (Table 2). Long-term BPV and baPWV were treated as continuous variables, adjusted for age, sex, smoking, drinking, physical activity, BMI, SBP, TC, TG, LDL-C, and FBG (model 4), baPWV was positively correlated with long-term BPV (β = 6.17, p < 0.001). The same held true for the three other multivariate models.

We conducted a sensitivity analysis repeating the multivariate regression analysis including only participants with complete data at the four observation times to avoid the impact of replacing missing values. There was a similar trend between the long-term BPV and baPWV (β = 6.56, p < 0.001). Moreover, to validate our findings, we repeated the above statistical analysis using the baPWV value from 2010 and calculating the SBPV using SBP from 2006, 2008, and 2010. This produced a similar result (Table 3).

## Discussion

The present study indicates that long-term SBPV is significantly associated with baPWV, even after adjusting for various confounding factors. We also validated this result using a sensitivity analysis. To the best of our knowledge, studies investigating the relationship between long-term SBPV and baPWV are rare.

Our findings are consistent with the literature. In untreated hypertensive outpatients, Ichihara *et al.* demonstrated that ambulatory mean BP levels and mean BPV were independently related to 24-h, daytime, and nighttime baPWV^20^. Another study found that nighttime SBPV was positively associated with carotid plaque^21^. It is possible that ambulatory BPV correlates with atherosclerosis. Our results suggest an increase of 6.16 cm/s in baPWV for every 1 mmHg increase in SBPV. Schillaci *et al.* suggested a 16 cm/s increase in baPWV for every 1 mmHg increase in 24-h SBPV^22^. Similarly, Ozawa *et al.* found that nighttime SBPV in patients with high BP was significantly positively associated with carotid-femoral PWV, with a standardized correlation coefficient of 0.228 (p < 0.05)^23^.

BPV is modulated by both physical and mental activity, as well as intrinsic cyclic vasomotion^24^. It is also inversely related to baroreceptor reflex sensitivity. The increase in BPV may be attributable to decreased compliance and increased stiffness of large elastic arteries caused by high BP^25^,^26^. The mechanism underlying this positive linear correlation between long-term SBPV and baPWV is unclear. Previous research suggests that the potential mechanism may involve the following. (1) Increased long-term BPV leads to an increase in the blood pressure fluctuation. This latter may increase vascular internal pressure, which damages the vascular intima and accelerates the process of atherosclerosis. (2) Increased long-term SBPV may also stimulate nerve-humoral mediation, inducing the expression of several inflammatory factors, such as TNF-α and IL-1. This promotes the proliferation of smooth muscle cell, accelerating the development of the atherosclerosis. (3) It’s also possible that increased baPWV leads to increased SBPV. Mattace-Raso *et al.* found that carotid arteriosclerosis reduced the sensitivity of the pressure sensor, which could lead to increased BPV^27^. However, we argued that increased BPV led to increased baPWV. Animal experiments have shown that long-term BPV increases while average blood pressure does not change after knocking out arterial baroreceptors in rats, and the increase of BPV led to an increased rate of arterial stiffness^28^,^29^,^30^.

This study must be interpreted whilst bearing in mind some limitations. First, the calculation of SBPV used four SBP values from annual physical examinations, which may affect the accuracy of SBPV. However, other studies have used only three annual SBP values^31^. Similarly, Shimbo *et al.* observed the factors influencing annual BPV in postmenopausal women, using just three annual BP values to calculate BPV^32^. In addition, we verified the finding by calculating the SBPV using BPV values from 2006, 2008, and 2010. Second, the measurement interval of BP was 2 years, which may affect the repeatability of SBPV. Nonetheless, previous studies have used intervals of between 1–3 years^33^,^34^. Furthermore, previous studies have used just one follow-up measurement, whereas we measured at three times^34^. This should ensure the repeatability of annual SBPV is higher. Finally, using average SBP to replace missing values in 2006, 2008, 2010, and 2012 may affect the accuracy of SBPV. However, our sensitivity analysis including only participants with complete data at the four times should avoid the impact of replacing data, and provided a similar result.

In conclusion, our results demonstrate that long-term BPV is significantly associated with arterial stiffness as assessed by baPWV. The evaluation of long-term BPV is important in the management of hypertensive patients, as well as the general population.

## Additional Information

**How to cite this article**: Wang, Y. *et al.* Association of long-term blood pressure variability and brachial-ankle pulse wave velocity: a retrospective study from the APAC cohort. *Sci. Rep.* **6**, 21303; doi: 10.1038/srep21303 (2016).

## Figures and Tables

**Table 1:** Demographic and baseline clinical characteristics of participants in different SBPV groups.

	SBPV (mmHg)				F/χ2	P-value
	Q1 (n = 998)	Q2 (n = 995)	Q3 (n = 1002)	Q4 (n = 999)		
Age (years)	48.16 ± 9.82	49.56 ± 10.49	50.46 ± 10.80	54.03 ± 11.53	52.71	<0.001
Male sex (%)	538 (53.9)	592 (59.5)	566 (56.5)	588 (58.9)	7.89	0.04
SBP (mmHg)	122.22 ± 14.41	124.16 ± 17.36	125.54 ± 18.47	131.17 ± 22.30	43.87	<0.001
BMI (kg/m^2^)	24.65 ± 3.33	24.88 ± 3.32	24.87 ± 3.30	25.14 ± 3.38	3.51	0.02
Lg TG	0.10 ± 0.26	0.11 ± 0.26	0.12 ± 0.26	0.15 ± 0.26	5.80	<0.01
TC (mmol/L)	4.85 ± 1.13	4.90 ± 1.12	5.03 ± 1.19	5.02 ± 1.03	5.67	<0.01
HDL-C (mmol/L)	1.52 ± 0.36	1.54 ± 0.41	1.55 ± 0.38	1.55 ± 0.39	1.19	0.31
FBG (mmol/L)	5.32 ± 1.40	5.24 ± 1.14	5.32 ± 1.35	5.59 ± 1.76	11.19	<0.001
Lg Hs-CRP	−0.19 ± 0.69	−0.18 ± 0.63	0.10 ± 0.64	0.06 ± 0.67	8.85	<0.001
Smoking (%)	271 (29.9)	313 (33.8)	327 (34.9)	306 (32.9)	5.79	0.12
Drinking (%)	342 (37.7)	377 (40.8)	369 (39.4)	347 (37.4)	2.89	0.41
Physical activity (%)	99 (20.7)	114 (23.8)	123 (25.7)	143 (29.9)	10.54	0.02
*Total*						
baPWV (cm/s)	1,492 ± 350	1,551 ± 405	1,580 ± 390	1,772 ± 429	84.57	<0.001
baPWV ≥ 1400 (%)	535 (46.4)	577 (58.0)	617 (61.6)	781 (78.2)	147.79	<0.001
*Left*						
baPWV (cm/s)	1,461 ± 340	1,516 ± 387	1,544 ± 377	1,725 ± 488	79.68	<0.001
baPWV ≥ 1400 (%)	484 (48.8)	538 (54.1)	586 (58.5)	734 (73.5)	141.54	<0.001
*Right*						
baPWV (cm/s)	1,455 ± 341	1,517 ± 391	1,541 ± 370	1,722 ± 471	83.38	<0.001
baPWV ≥ 1400 (%)	488 (48.9)	547 (55.0)	580 (57.9)	756(75.7)	153.02	<0.001

SBP: systolic blood pressure; BMI: Body Mass Index; Lg TG: triglyceride after logarithmic transformation; TC: total cholesterol; HDL-C: high density lipoprotein cholesterol; FBG: fasting blood-glucose; Lg Hs-CRP: C-reactin protein after logarithmic transformation; SBPV: systolic blood pressure variability; baPWV: Brachial-ankle pulse wave velocity.

**Table 2:** Multivariate linear regression analysis of the influence of long-term BPV (measured in 2006, 2008, 2010 and 2012) on brachial-ankle pulse wave velocity.

	β	S.E.	95% CI	P-value
Model 1	16.91	1.10	14.76–19.07	<0.001
Model 2	9.94	0.97	8.04–11.85	<0.001
Model 3	7.29	0.95	5.42–9.16	<0.001
Model 4	6.17	0.99	4.24–8.12	<0.001
Model 4*	6.56	0.98	4.64–8.48	<0.001

β: partial regression coefficient; SE: standard error; 95% CI: 95% confidence interval.

Model 1: unadjusted;

Model 2: adjusted for age and sex;

Model 3: adjusted for age, sex, smoking, drinking, physical activity, and body mass index;

Model 4: adjusted for age, sex, smoking, drinking, physical activity, body mass index, systolic blood pressure, total cholesterol, triglyceride, low density lipoprotein cholesterol, and fasting blood glucose.

Model 4* (sensitivity analysis): adjusted for age, sex, smoking, drinking, physical activity, body mass index, systolic blood pressure, total cholesterol, triglyceride, low density lipoprotein cholesterol, and fasting blood glucose.

**Table 3:** Multivariate linear regression analysis of the influence of long-term BPV (measured in 2006, 2008, and 2010) on brachial-ankle pulse wave velocity.

	β	S.E.	95% CI	P-value
Model 1	13.89	0.76	12.40–15.39	<0.001
Model 2	8.60	0.62	7.39–9.82	<0.001
Model 3	6.58	0.58	5.45–7.71	<0.001
Model 4	6.14	0.59	4.98–7.29	<0.001

β: partial regression coefficient; SE: standard error; 95% CI: 95% confidence interval.

Model 1: unadjusted;

Model 2: adjusted for age and sex;

Model 3: adjusted for age, sex, smoking, drinking, physical activity, body mass index;

Model 4: adjusted for age, sex, smoking, drinking, physical activity, body mass index, systolic blood pressure, total cholesterol, triglyceride, low density lipoprotein cholesterol, fasting blood glucose.

## References

[b1] Frattola A., Parati G., Cuspidi C., Albini F. & Mancia G. Prognostic value of 24-hour blood pressure variability. J Hypertens 11, 1133–1137 (1993). 8258679 10.1097/00004872-199310000-00019

[b2] Kikuya M. *et al.* Prognostic significance of blood pressure and heart rate variabilities: the Ohasama study. Hypertension 36, 901–906 (2000). 11082164 10.1161/01.hyp.36.5.901

[b3] Verdecchia P. *et al.* Prognostic significance of blood pressure variability in essential hypertension. Blood Press Monit 1, 3–11 (1996). 10226196

[b4] Parati G., Ochoa J. E., Lombardi C. & Bilo G. Assessment and management of blood-pressure variability. Nat Rev Cardiol 10, 143–155 (2013). 23399972 10.1038/nrcardio.2013.1

[b5] Rothwell P. M. *et al.* Prognostic significance of visit-to-visit variability, maximum systolic blood pressure, and episodic hypertension. Lancet 375, 895–905 (2010). 20226988 10.1016/S0140-6736(10)60308-X

[b6] Tsioufis C. *et al.* Comparative prognostic role of nighttime blood pressure and nondipping profile on renal outcomes. Am J Nephrol 33, 277–288 (2011). 21372563 10.1159/000324697

[b7] Seo W. W. *et al.* The value of brachial-ankle pulse wave velocity as a predictor of coronary artery disease in high-risk patients. Korean Circ J 40, 224–229 (2010). 20514332 10.4070/kcj.2010.40.5.224 PMC2877786

[b8] Ichihara A. *et al.* Low doses of losartan and trandolapril improve arterial stiffness in hemodialysis patients. Am J Kidney Dis 45, 866–874 (2005). 15861352 10.1053/j.ajkd.2005.02.022

[b9] Yamashina A. *et al.* Validity, reproducibility, and clinical significance of noninvasive brachial-ankle pulse wave velocity measurement. Hypertens Res 25, 359–364 (2002). 12135313 10.1291/hypres.25.359

[b10] Ait-Oufella H. *et al.* Long-term reduction in aortic stiffness: a 5.3-year follow-up in routine clinical practice. J Hypertens 28, 2336–2341 (2010). 20683338 10.1097/HJH.0b013e32833da2b2

[b11] Boutouyrie P. *et al.* Aortic stiffness is an independent predictor of primary coronary events in hypertensive patients: a longitudinal study. Hypertension 39, 10–15 (2002). 11799071 10.1161/hy0102.099031

[b12] Asmar R. G. *et al.* Improvement in blood pressure, arterial stiffness and wave reflections with a very-low-dose perindopril/indapamide combination in hypertensive patient: a comparison with atenolol. Hypertension 38, 922–926 (2001). 11641310 10.1161/hy1001.095774

[b13] Amar J., Ruidavets J. B., Chamontin B., Drouet L. & Ferrieres J. Arterial stiffness and cardiovascular risk factors in a population-based study. J Hypertens 19, 381–387 (2001). 11288807 10.1097/00004872-200103000-00005

[b14] Ichihara A. *et al.* Long-term effects of intensive blood-pressure lowering on arterial wall stiffness in hypertensive patients. Am J Hypertens 16, 959–965 (2003). 14573335 10.1016/s0895-7061(03)01004-5

[b15] Wang A. *et al.* Resting heart rate and risk of cardiovascular diseases and all-cause death: the Kailuan study. PLoS One 9, e110985 (2014). 25343354 10.1371/journal.pone.0110985 PMC4208799

[b16] Zhou Y. *et al.* Asymptomatic polyvascular abnormalities in community (APAC) study in China: objectives, design and baseline characteristics. PLoS One 8, e84685 (2013). 24386406 10.1371/journal.pone.0084685 PMC3873465

[b17] Wang A. *et al.* Measures of adiposity and risk of stroke in China: a result from the Kailuan study. PLoS One 8, e61665 (2013). 23613897 10.1371/journal.pone.0061665 PMC3629147

[b18] Pringle E. *et al.* Systolic blood pressure variability as a risk factor for stroke and cardiovascular mortality in the elderly hypertensive population. J Hypertens 21, 2251–2257 (2003). 14654744 10.1097/00004872-200312000-00012

[b19] Tatasciore A. *et al.* Awake systolic blood pressure variability correlates with target-organ damage in hypertensive subjects. Hypertension 50, 325–332 (2007). 17562971 10.1161/HYPERTENSIONAHA.107.090084

[b20] Ichihara A., Kaneshiro Y., Takemitsu T., Sakoda M. & Hayashi M. Ambulatory blood pressure variability and brachial-ankle pulse wave velocity in untreated hypertensive patients. J Hum Hypertens 20, 529–536 (2006). 16598288 10.1038/sj.jhh.1002023

[b21] Shintani Y. *et al.* Ambulatory blood pressure, blood pressure variability and the prevalence of carotid artery alteration: the Ohasama study. J Hypertens 25, 1704–1710 (2007). 17620969 10.1097/HJH.0b013e328172dc2e

[b22] Schillaci G. *et al.* Relationship between short-term blood pressure variability and large-artery stiffness in human hypertension: findings from 2 large databases. Hypertension 60, 369–377 (2012). 22753222 10.1161/HYPERTENSIONAHA.112.197491

[b23] Ozawa M. *et al.* Blood pressure variability as well as blood pressure level is important for left ventricular hypertrophy and brachial-ankle pulse wave velocity in hypertensives. Clin Exp Hypertens 31, 669–679 (2009). 20001459 10.3109/10641960903407033

[b24] Pickering T. G. Variability of blood pressure. Blood Press Monit 3, 141–145 (1998). 10212344

[b25] Imai Y. *et al.* Factors that affect blood pressure variability. A community-based study in Ohasama, Japan. Am J Hypertens 10, 1281–1289 (1997). 9397248 10.1016/s0895-7061(97)00277-x

[b26] Mancia G. & Grassi G. Mechanisms and clinical implications of blood pressure variability. J Cardiovasc Pharmacol 35, S15–19 (2000). 11346215 10.1097/00005344-200000004-00003

[b27] Mattace-Raso F. U. *et al.* Arterial stiffness, cardiovagal baroreflex sensitivity and postural blood pressure changes in older adults: the Rotterdam Study. J Hypertens 25, 1421–1426 (2007). 17563564 10.1097/HJH.0b013e32811d6a07

[b28] Sasaki S. *et al.* Association of blood pressure variability with induction of atherosclerosis in cholesterol-fed rats. Am J Hypertens 7, 453–459 (1994). 8060580 10.1093/ajh/7.5.453

[b29] Lacolley P. *et al.* Aortic distensibility and structural changes in sinoaortic-denervated rats. Hypertension 26, 337–340 (1995). 7635544 10.1161/01.hyp.26.2.337

[b30] Lacolley P. *et al.* Structural changes and *in situ* aortic pressure-diameter relationship in long-term chemical-sympathectomized rats. Am J Physiol 269, H407–416 (1995). 7653604 10.1152/ajpheart.1995.269.2.H407

[b31] Muntner P. *et al.* The relationship between visit-to-visit variability in systolic blood pressure and all-cause mortality in the general population: findings from NHANES III, 1988 to 1994. Hypertension 57, 160–166 (2011). 21200000 10.1161/HYPERTENSIONAHA.110.162255

[b32] Shimbo D. *et al.* Association between annual visit-to-visit blood pressure variability and stroke in postmenopausal women: data from the Women’s Health Initiative. Hypertension 60, 625–630 (2012). 22753206 10.1161/HYPERTENSIONAHA.112.193094 PMC3427141

[b33] Chen W. *et al.* Low birth weight is associated with higher blood pressure variability from childhood to young adulthood: the Bogalusa Heart Study. Am J Epidemiol 176 Suppl 7, S99–105 (2012). 23035149 10.1093/aje/kws298 PMC3530367

[b34] Howard S. C. & Rothwell P. M. Reproducibility of measures of visit-to-visit variability in blood pressure after transient ischaemic attack or minor stroke. Cerebrovasc Dis 28, 331–340 (2009). 19628934 10.1159/000229551

